# Enterovirus D68 Infection, Chile, Spring 2014

**DOI:** 10.3201/eid2104.141766

**Published:** 2015-04

**Authors:** Juan P. Torres, Mauricio J. Farfan, Giannina Izquierdo, Paula Piemonte, Juan Henriquez, Miguel L. O’Ryan

**Affiliations:** Clinica Las Condes, Santiago, Chile (J.P. Torres, M.J. Farfan, G. Izquierdo, P. Piemonte, J. Henriquez, M.L. O’Ryan);; University of Chile, Santiago (J.P. Torres, M.J. Farfan, G. Izquierdo, M.L. O’Ryan)

**Keywords:** enterovirus 68, enterovirus D68, status asthmaticus, Chile, viruses

**To the Editor:** Enterovirus D68 (EV-D68) is an emergent viral pathogen associated with severe respiratory illness, especially in children with asthma (*1*). The ongoing epidemic in the United States has expanded to 47 states; as of November 25, 2014, a total of 1,121 persons were affected (http://www.cdc.gov/non-polio-enterovirus/outbreaks/EV-D68-outbreaks.html). 

In Chile, because of clinical suspicion of infection caused by the same EV-D68 reported in the United States, we conducted further testing. We selected 6 children who were at Clinica Las Condes (CLC), Santiago, during September–October 2014, for whom nasopharyngeal samples were enterovirus positive according to multiplex PCR (CLART PneumoVir; Genomica, Madrid, Spain) or rhinovirus/enterovirus positive according to the FilmArray Respiratory Panel (BioFire Diagnostics, Salt Lake City, UT, USA). The CLC ethics committee authorized the study. CLC is the second largest private hospital in eastern Santiago, which has a population of nearly 400,000. Since 2008, detection of respiratory viruses by CLART PneumoVir has been performed for all hospitalized CLC patients with respiratory disease. This test detects 17 respiratory viruses, including enterovirus (generic detection). Since 2014, CLC also incorporated testing with the FilmArray Respiratory Panel, which does not distinguish enterovirus from rhinovirus. 

We sent 6 nasopharyngeal samples from the children to the US Centers for Disease Control and Prevention for detection of EV-D68 by real-time reverse transcription PCR (http://www.cdc.gov/non-polio-enterovirus/downloads/ev-d68-rt-pcr-protocol.pdf) and sequencing (*2*). Of the 6 patients, EV-D68 was confirmed for 2 patients.

Patient 1 was a boy, 7.5 years of age, who was hospitalized on September 21, 2014, with a history of asthma since 3 years of age. During the previous 3 months, his father had frequently traveled to the United States. The patient’s current episode began with upper respiratory symptoms and low-grade fever (38°C) and was followed by intense vomiting. On admission, the child exhibited respiratory distress and an oxygen saturation of 88%, which led to his admission to the pediatric intensive care unit and management with noninvasive mechanical ventilation (bilevel positive airway pressure) for 48 hours. He was discharged home after 5 days of hospitalization, having required supplemental oxygen for 4 of those days.

Patient 2 was a 9-year-old boy who was hospitalized on October 1, 2014, with a history of severe asthma since 7 years of age. He had visited the emergency department multiple times for asthma crises. Both parents and his 2 brothers also had asthma. The episode reported here began 4 days before hospitalization, with a dry cough and progressive breathing difficulty, requiring 3 emergency department visits. During the third visit, low oxygen saturation (91%) led to hospitalization. The child had no fever during this entire episode. He received up to 50% fraction of inspired oxygen, intravenous methylprednisolone, intravenous magnesium sulfate, and inhaled salbutamol; he was discharged in good medical condition after 8 days of hospitalization, having received supplemental oxygen for 7 of those days.

These 2 EV-D68–positive patients had marked pulmonary hyperinsufflation and required prolonged oxygen therapy. Sequence analysis of the viral protein 1 gene revealed that both of these viruses clustered with the major outbreak strain from the United States. Partial gene sequences of viral protein 1 were deposited in the GenBank database under accession nos. KP247599 and KP247600.

We next used CLART PneumoVir to retest samples that had been positive for enterovirus/rhinovirus by FilmArray during September–October 2013 and 2014. The number of overall samples tested for respiratory viruses did not increase from 2013 to 2014 (227 and 218, respectively), but the percentage of enteroviruses detected increased strikingly (from 2.6% to 14.6%). We then compared clinical characteristics and their frequency of occurrence among enterovirus-positive patients hospitalized during September–October 2013 and September–October 2014. The clinical features of 24 enterovirus-positive patients hospitalized during 2014 differed from those of 5 enterovirus-positive children hospitalized during 2013. Hospitalization in 2014 was mostly for asthmatic crisis in children 2.5 to 7 years of age; this pattern is less clear for the few patients hospitalized in 2013 (Table). A substantial proportion of patients hospitalized in 2014 required oxygen support and admission to the pediatric intensive care unit.

**Table:** Clinical features of enterovirus–positive patients, Chile, September–October 2013 and 2014*

Feature	2013, n = 6	2014, n = 27
Median age, y (IQR)	1.6 (0.25–5.0)	6.0 (2.5–7.0)
Male sex, %	50	52
Admitted to hospital, no.	5	24
Admission diagnosis, no. (%)		
Asthmatic crisis	1 (20)	16 (67)†
Obstructive bronchitis	2 (40)	5 (21)
Pneumonia	0	2 (8)
Other‡	2 (40)	1 (4)
Asthma as preexisting condition, no. (%)	1 (20)	13 (54)
Median oxygen saturation at admission (IQR)	96 (95.0–98.5)	91 (88.5–93.5)
Admitted to PICU, median no. (%)	0	9 (37)
Ventilation support required, no. (%)	0	5 (21)
Median days requiring supplemental oxygen (IQR)	0 (1–4)	2.5 (1–4.5)
Median days in hospital (IQR)§	3 (3–7)	4 (4–6.5)

*IQR, interquartile range; PICU, pediatric intensive care unit. †p = 0.07 when considering only hospitalized and p = 0.05 when considering all 6 and 27 enterovirus-positive children, by 1-tailed Fisher exact test. ‡Febrile with seizure (n = 1), fever of unknown origin (n = 1), convulsion in child with epilepsy (n = 1). §In 2014, one child was transferred to another hospital, and duration of hospital stay is unknown.

In conclusion, we report 2 confirmed cases of EV-D68 in a Southern Hemisphere country during the 2014 outbreak reported in the United States. That these cases are virologically and clinically related to those reported in the United States documents that the virus had been introduced to the Southern Hemisphere during the spring of 2014. A substantial increase in enterovirus cases displaying a notably similar clinical pattern (asthmatic crisis in children) strongly suggests that EV-D68 infections are increasingly rapidly. This virus has been previously identified in the region (*3*) but only sporadically. The virus could spread to other areas in Santiago and to other cities, and similar situations could occur in other Latin American countries, especially those with many residents who travel to the United States. Public health officials need to be notified of this potential, and appropriate surveillance and treatment strategies need to be implemented.
